# RAC1 plays an essential role in estrogen receptor alpha function in breast cancer cells

**DOI:** 10.1038/s41388-021-01985-1

**Published:** 2021-08-09

**Authors:** Jun Sun, Gabriel Gaidosh, Ye Xu, Adnan Mookhtiar, Na Man, Pradeep Reddy Cingaram, Ezra Blumenthal, Ramin Shiekhattar, Erik T. Goka, Stephen D. Nimer, Marc E. Lippman

**Affiliations:** 1 Department of Medicine, University of Miami Miller School of Medicine, Miami, FL USA; 2 Sylvester Comprehensive Cancer Center, University of Miami Miller School of Medicine, Miami, FL USA; 3 Department of Human Genetics, University of Miami, Miami, FL USA; 4 Geneyus, LLC, Miami, FL USA; 5 Department of Biochemistry and Molecular Biology, University of Miami, Miami, FL USA; 6 Lombardi Comprehensive Cancer Center, Georgetown University Medical Center, Washington, DC USA

**Keywords:** Breast cancer, Hormone receptors

## Abstract

The activity of Rho family GTPase protein, RAC1, which plays important normal physiological functions, is dysregulated in multiple cancers. RAC1 is expressed in both estrogen receptor alpha (ER)-positive and ER-negative breast cancer (BC) cells. However, ER-positive BC is more sensitive to RAC1 inhibition. We have determined that reducing RAC1 activity, using siRNA or EHT 1864 (a small molecule Rac inhibitor), leads to rapid ER protein degradation. RAC1 interacts with ER within the ER complex and RAC1 localizes to chromatin binding sites for ER upon estrogen treatment. RAC1 activity is important for RNA Pol II function at both promoters and enhancers of ER target genes and ER-regulated gene transcription is blocked by EHT 1864, in a dose-dependent manner. Having identified that RAC1 is an essential ER cofactor for ER protein stability and ER transcriptional activity, we report that RAC1 inhibition could be an effective therapeutic approach for ER-positive BC.

## Introduction

Estrogens play important physiological roles in various organs and participate in cancer development [1], particularly in the 80% of breast cancers that are estrogen receptor alpha (ER)-positive [2], where estrogen promotes tumor growth [3]. ER is activated by estrogen to regulate gene transcription [4]. Estrogen stimulates the assembly of a multi-protein complex, which contains co-activators such as steroid receptor coactivators, and chromatin remodelers such as CARM1 and p300/CBP that cooperatively regulate ER target gene expression [5–8].

Rac (The Ras-related C3 botulinum toxin substrate) is a subfamily of the Rho family of GTPases [9]. Three Rac subclass proteins, RAC1, RAC2 and RAC3, share over 90% identity, but differ in their patterns of expression. RAC1 is ubiquitously expressed [10] and plays important roles in cell adhesion, migration, cell cycle progression, transcriptional regulation, and tumor formation. RAC1 cycles between a GDP-bound inactive form and a GTP-bound active form and is activated by multiple mechanisms in various cancers [11–13]. Many signaling molecules, including p21-activated kinases (PAKs), LIM kinase, PI3K or mitogen-activated protein kinases (MAPKs) are effector proteins of active RAC1 [14]. RAC1 activating mutations have been found in several cancers [15, 16]. Breast cancer has higher RAC1 expression than normal breast tissue [17], which can be activated through EGFR family members [18]. We have shown that loss of HACE1, an E3 ubiquitin ligase for RAC1, leads to enhanced RAC1 signaling that contributes to breast cancer development [19]. Small molecule Rac inhibitors have been developed as potential cancer therapeutic agents [20–22], such as the Rac inhibitor EHT 1864, which specifically blocks Rac and GTP interaction and keeps Rac in an inactive state [23].

ER-positive breast cancer patients have been successfully treated with endocrine therapies, such as SERMs that block ER function, or aromatase inhibitors that limit the production of estrogen [24]. However, de novo or acquired resistance to endocrine therapies remain challenging clinical problems [25]. Combining endocrine therapies with various targeted approaches, such as CDK4/6 inhibitors, have been evaluated as strategies to overcome endocrine resistance [26, 27]. Novel treatment strategies to overcome endocrine resistance are needed.

We found that the proliferation of ER-positive breast cancer cells is more sensitive to Rac inhibition than ER-negative breast cancer cells that is consistent with earlier findings [28, 29], and that RAC1 is an intrinsic and essential component of the ER complex. RAC1 activity is required for ER protein stability and estrogen-stimulated ER transcriptional activity in ER+ BC, likely explaining why the Rac inhibitor EHT 1864 represses ER target genes and is potentially helpful in combating endocrine resistance [28], utilizing a mechanism distinct from the action of anti-estrogens. Thus, RAC1 inhibitor suitable for clinical use may be a promising agent for treating endocrine-resistant breast cancer.

## Results

### ER-positive breast cancer cell growth is sensitive to RAC1 inhibition

**Fig. 1:**
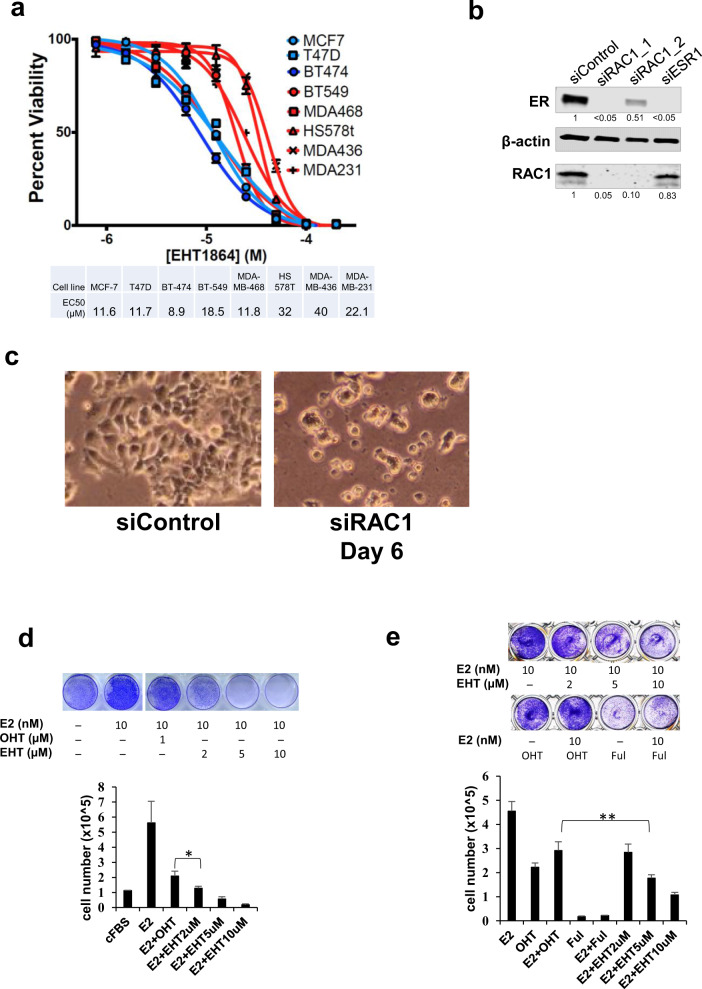
RAC1 is essential for ER-positive breast cancer cell growth. **A** Cell viability was measured by PrestoBlue after the cells were treated with different dosages of the RAC1 inhibitor EHT 1864 for three days. Among the tested breast cancer cell lines, MCF-7, T47D, BT-474 are ER-positive breast cancer cells; BT-549, HS578T, MDA-MB-468, MDA-MB-436, and MDA-MB-231 are ER-negative breast cancer cells. **B** The Western blot analyses for changes in RAC1 or ER protein levels three days after MCF-7 cells were transfected with siRNA oligos. Quantification of protein targets was measured with Image Studio (Li-COR) after normalizing to β-actin and setting targets in the control sample as 1. **C** Extended down-regulation of RAC1 in MCF-7 cells led to cell death. MCF-7 cells were transfected with non-targeted siRNA (siControl) or si*RAC1* on day 0 and day 3. The microscopic images were taken on day 6. **D** MCF-7 cells or (**E**) LCC2 cells were seeded overnight with 20k cells per well in a 24-well plate in estrogen-deprivation media and then treated with E2, OHT (1 μM), Fulvestrant (1 μM) or EHT1864 as indicated for 5 days or 7 days, respectively. The representative pictures of crystal violet staining are shown at the top. The cells were counted from 3 independently treated samples. The data are presented as means ± SD. *P = 0.015; **P = 0.007 (Student’s *t* test).

RAC1 is ubiquitously expressed in both ER-positive and ER-negative breast cancer cells [30]. We found that ER-positive breast cancer cells were more readily inhibited by the Rac inhibitor, EHT 1864, than ER-negative breast cancer cells (Fig. 1A), consistent with published results [28, 29]. We found that protein levels of RAC1 and ER were reduced in MCF-7 cells after cells were transfected with siRNAs targeting *RAC1*, although two different siRNA oligos had different efficiencies in reducing ER protein (Fig. 1B). Similar results were found in another ER-positive breast cancer cell line T47D (Fig. S1A). We observed cell death and detachment by day 6 after the extended siRAC1 treatment (Fig. 1C), suggesting RAC1 is required for survival of MCF-7 cells. However, in ER-negative MDA-MB-468 cells, we were able to knock out *RAC1* using CRISPR-Cas9 based gene editing technique and maintain the cell growth (Fig. S1B), indicating the loss of RAC1 can be compensated in this cell type.

We treated estrogen-deprived MCF-7 cells with EHT 1864, and found that 2 µM EHT 1864 was more effective than 4-hydroxytamoxifen (OHT) in blocking E2-stimulated MCF-7 cell growth; 5 or 10 μM EHT 1864 prevented all E2-stimulated cell growth (Fig. 1D). Using tamoxifen-resistant LCC2 cells [31], we found that 5 μM or higher EHT 1864 were effective in inhibiting cell growth (Fig. 1E). Thus, RAC1 inhibition holds potential to be a therapeutic approach in treating ER+ BC.

### RAC1 is essential for ER-regulated transcription and ER protein stability

**Fig. 2:**
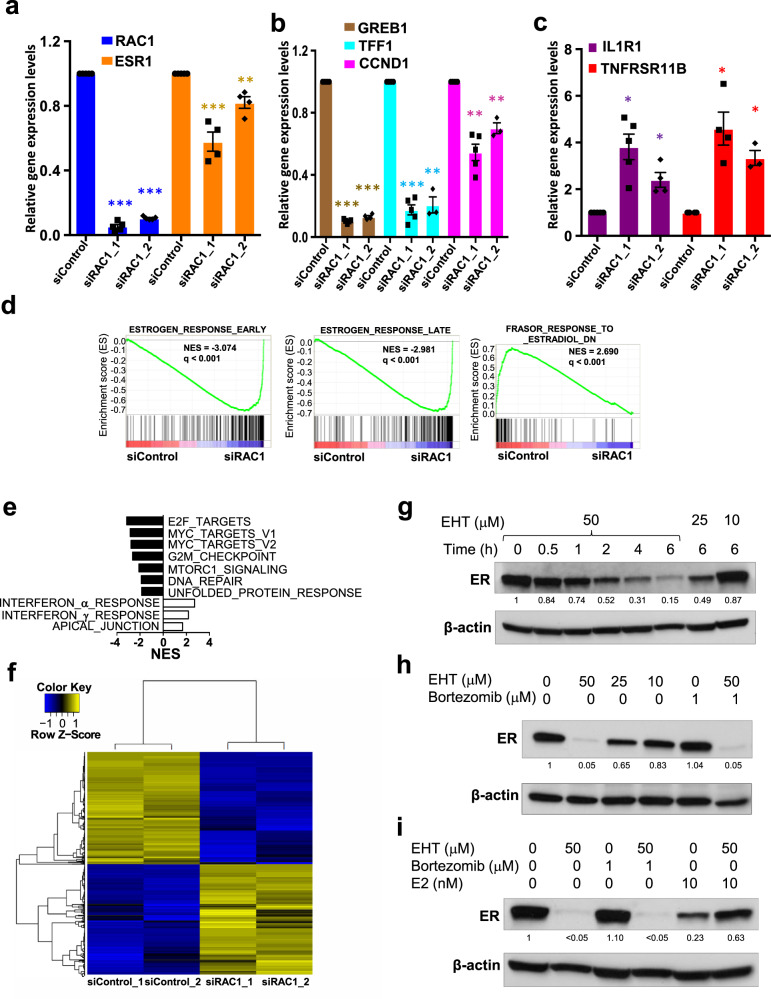
RAC1 is required for ER activity. MCF-7 cells were grown in estrogen-containing media and were transfected with either non-targeting siRNA or two different siRNAs targeting *RAC1*. The cells were collected after three days for RT-qPCR assay for the indicated targets: *RAC1* and *ESR1* (**A**); ER-activated genes *GREB1*, *TFF1* and *CCND1* (**B**); ER-repressed genes *IL1R1* and *TNFRSF11B* (**C**). The data are presented from 3 to 5 independent transfection assays shown as mean ± SEM. Student’s *t* tests were performed by comparing gene expression levels in cells transfected with siRAC1 to cells transfected with siControl: *P < 0.005; **P < 0.001; ***P < 10^−4^. **D** RNA-seq analyses were performed from two biological replicate RNA samples prepared from MCF-7 cells after the cells were transfected with siRAC1 or control siRNA oligo for three days. GSEA analysis indicated significant repression of ER-activated target genes, but activation of ER-repressed target genes upon down-regulating RAC1. **E** Some other hallmark pathways were significantly changed after RAC1 down-regulation in MCF-7 cells. **F** The heatmap shows the genes with 2 fold changes (FDR < 0.05) in MCF-7 cells after transfection with siRAC1 for 3 days. **G** The Western blot analysis for ER protein in MCF-7 cells grown in the estrogen-containing media treated with different dosages of the RAC1 inhibitor EHT1864 at the indicated time points; or after 6 h treatment with combination of EHT1864 and proteasome inhibitor Bortezomib (**H**). **I** The Western blot analysis for ER protein in MCF-7 cells that were maintained in the estrogen-deprived media for three days followed by the indicated treatments for 6 h. The target protein was quantified as in Fig. 1B.

Given the growth inhibitory effects of *RAC1* mRNA knockdown on MCF-7 cells, we looked for changes in ER target gene expression using RT-qPCR. *RAC1* mRNA levels were reduced by about 95% or 90% with two different siRAC1 oligos, respectively (Fig. 2A), consistent with the decrease in RAC1 protein (Fig. 1B). In contrast, *ESR1* mRNA levels were modestly reduced (Fig. 2A), but ER protein levels were reduced much more significantly (Fig. 1B). The expression of several canonical ER-activated genes, such as *GREB1*, and *TFF1*, was also greatly reduced while CCND1 had modest reduction (Fig. 2B). Expression of ER-repressed genes, such as *IL1R1* and *TNFRSF11B*, were up-regulated by siRAC1 (Fig. 2C). Consistently, the more efficient siRAC1 oligo 1 had greater effects than the slightly less efficient siRAC1 oligo 2 (Fig. 2A–C).

We performed RNA-seq analysis in MCF-7 cells three days after the cells were transfected with the more efficient siRAC1 oligo. GSEA analysis indicated down-regulation of both early and late ER target genes, but up-regulation of estrogen down-regulated genes [32] (Fig. 2D). Other hallmark pathways, such as MYC and E2F targets, were significantly down-regulated; interferon response and apical junction genes were significantly up-regulated (Fig. 2E). Overall, we identified 2251 genes with >2-fold differences in expression levels (FDR < 0.05) after down-regulation of RAC1 (Supplementary Table 1). The numbers of up-regulated or down-regulated genes are about equally distributed (Fig. 2F).

In MCF-7 cells, RAC1 is the dominant RAC family member expressed. *RAC1* mRNA is about 9 fold higher than *RAC3* mRNA. *RAC2* is not expressed (Fig. S1C, D). Most ER protein (>95%) disappeared 24 h after transfecting siRNA oligo targeting *ESR1* (Fig. S1E). ER protein was reduced by close to 90%, and more at 48 h, in si*RAC1*-transfected cells, but was not significantly changed in si*RAC3*-transfected cells (Fig. S1E). Specific down-regulation of RAC3 did not repress the expression of canonical ER target genes, such as *GREB1* or *TFF1* (Fig. S1F). We consistently observed slower ER protein reduction in MCF-7 cells after down-regulating *RAC1* than in cells after down-regulating *ESR1*, suggesting siRAC1-induced ER protein reduction may happen at the post-transcriptional level.

We treated proliferating MCF-7 cells with EHT 1864 for various durations up to 6 h. Within 30 min, ER protein levels started to fall (Fig. 2G), following exposure to EHT 1864 at 50 μM, a concentration sufficient to completely inhibit RAC1 activity [21]. Twenty five μM ETH 1864 degraded ER protein at a slower rate (Fig. 2G), while lower concentrations of EHT 1864 (10 μM or lower) that only partially inhibit RAC1, did not reduce ER protein levels (Fig. 2G). Thus, the ER protein level depends on the RAC1 protein level and activity. Unlike estrogen-stimulated ER degradation [33], EHT 1864-induced ER degradation is not mediated by the proteasome pathway, as the proteasome inhibitor Bortezomib did not block ER degradation (Fig. 2H). While ER protein is stable in MCF-7 cells in absence of estrogen, E2 triggers the proteasomal degradation of ER protein [34]. We compared estrogen-induced to EHT1864-induced ER degradation (Fig. 2I) and found that 50 μM EHT 1864 degraded ER faster and more completely than E2 alone. E2 actually antagonized the degradation of ER triggered by EHT 1864 (Fig. 2I), suggesting they involve divergent degradation pathways. Chloroquine, an autophagy inhibitor, failed to block EHT 1864-induced ER degradation, suggesting that lysosomes are not involved in ER degradation (Fig. S1G). While the mechanism involved in EHT 1864 mediated ER degradation remains elusive, EHT 1864-induced ER degradation appears to be similar to that induced by Hsp90 inhibitors [35], which is also not blocked by proteasome inhibitors. Nonetheless, RAC1 activity appears to be critical to maintaining ER protein levels in ER+ breast cancer cells.

**Fig. 3:**
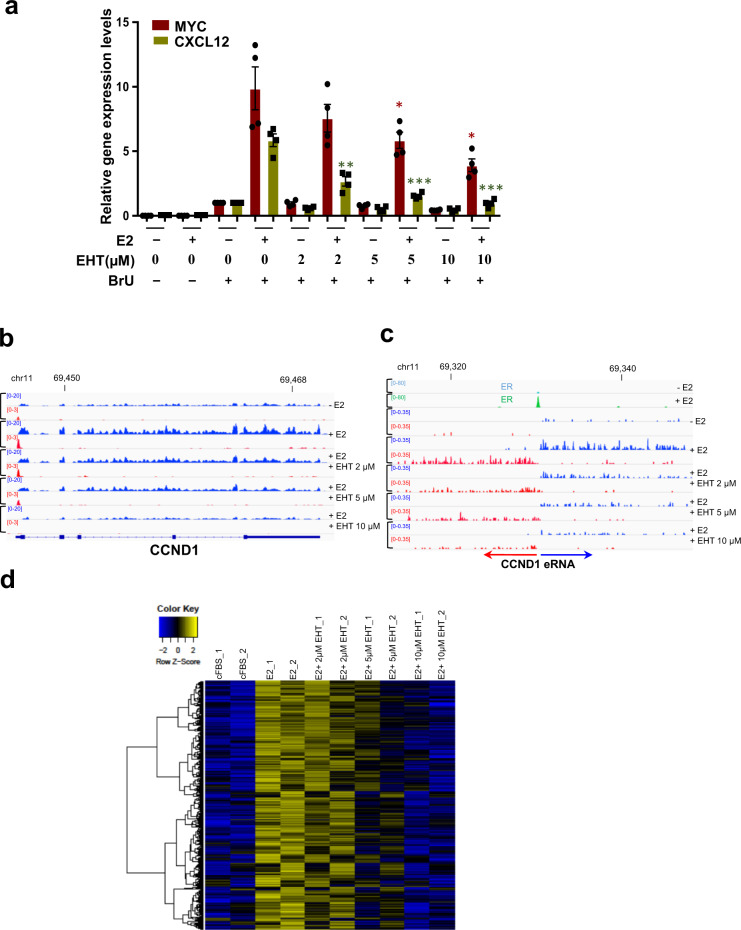
RAC1 inhibitor blocks ER transcriptional activity. **A** MCF-7 cells were deprived of estrogen for three days before 1 h treatment with 10 nM E2 or different dosages of EHT 1864. Two mM BrU was added to the culture at the start of the treatment. Total RNA was extracted and subjected to two rounds of immunoprecipitation with an antibody against BrdU. cDNA was generated and subjected to qPCR analysis for the ER target genes *MYC* and *CXCL12*. Their basal expression levels in the absence of E2 were set as 1. Data are presented as mean ± SD from 4 tests using two independent RNA-IP samples. *P < 0.05; **P < 0.002; ***P < 10^−4^ (Student’s *t* test). **B**–**D** RNA-seq analyses were performed using the RNA templates purified from two independent IP as in (**A**). Genome browser snapshots are shown for RNA expression at *CCND1* locus (**B**) or at a *CCND1* enhancer site occupied by ER (**C**) when MCF-7 cells were treated with E2 or in combination with EHT 1864 at the indicated concentrations. Blue or red track indicates RNA transcripts derived from sense or anti-sense strand, respectively. The top 2 tracks in (**C**) display ChIP-seq signal profiles for ER in the absence or presence of E2. **D** Heatmap for genes with greater than 2-fold (adjusted *P* < 0.05) increase after 1 h E2 treatment. These genes were inhibited by EHT 1864 in a dose dependent manner.

To measure the direct effects of Rac inhibition on ER-regulated transcription, we treated estrogen-deprived MCF-7 cells with E2 and different concentrations of EHT 1864 (2, 5, or 10 µM) for 1 h, during which the newly synthesized RNAs were labeled with 5-bromouridine (BrU) [36]. We performed RT-qPCR analyses on two ER target genes *MYC* and *CXCL12*, from the newly synthesized RNAs purified with an antibody against BrdU while RNA samples from cells not treated with BrU were included as negative controls (Fig. 3A). E2 strongly induced these two genes in 1 h. EHT 1864 reduced E2-stimulated transcription in a dose-dependent manner. Very little non-labeled RNAs were purified with this approach (Fig. 3A). We performed RNA-seq analyses on these newly synthesized RNAs (BrU-seq). Examples are shown for *CCND1* at its gene locus (Fig. 3B) and enhancer site (Fig. 3C). Both the coding transcripts of *CCND1* and the antisense transcripts upstream of *CCND1* promoter were significantly increased upon estrogen treatment. And they were reduced by elevating dosages of EHT 1864. These indicate EHT 1864 impairs estrogen-stimulated *CCND1* promoter activity. Distant upstream to *CCND1* lies an ER-bound enhancer that was well characterized [37]. Estrogen simulated ER binding to this enhancer and transcription of bi-directional enhancer RNAs that were started from the center of this ER binding site (Fig. 3C). Similarly, EHT 1864 repressed the biosynthesis of these estrogen-induced enhancer RNAs in a dose-dependent manner (Fig. 3C). Thus, this ER-bound *CCND1* enhancer function was attenuated by EHT 1864. The reduction of ER transcriptional activity is not due to decreased ER protein levels since EHT 1864 at 10 μM or less did not reduce ER protein (Fig. 2G). Therefore, RAC1 modulates both ER transcriptional activity and protein stability via distinct mechanisms.

We detected 731 genes with >2 fold changes (adjusted *p* < 0.05) (Supplementary Table 2) after 1 h E2 treatment. Among them, 629 genes were up-regulated and 102 genes were down-regulated. EHT 1864 did not significantly reverse E2-induced gene repression. However, E2 up-regulated genes were significantly inhibited by escalating dosages of EHT 1864 (Fig. 3D).

### RAC1 is a component in the ER complex in breast cancer cells

**Fig. 4:**
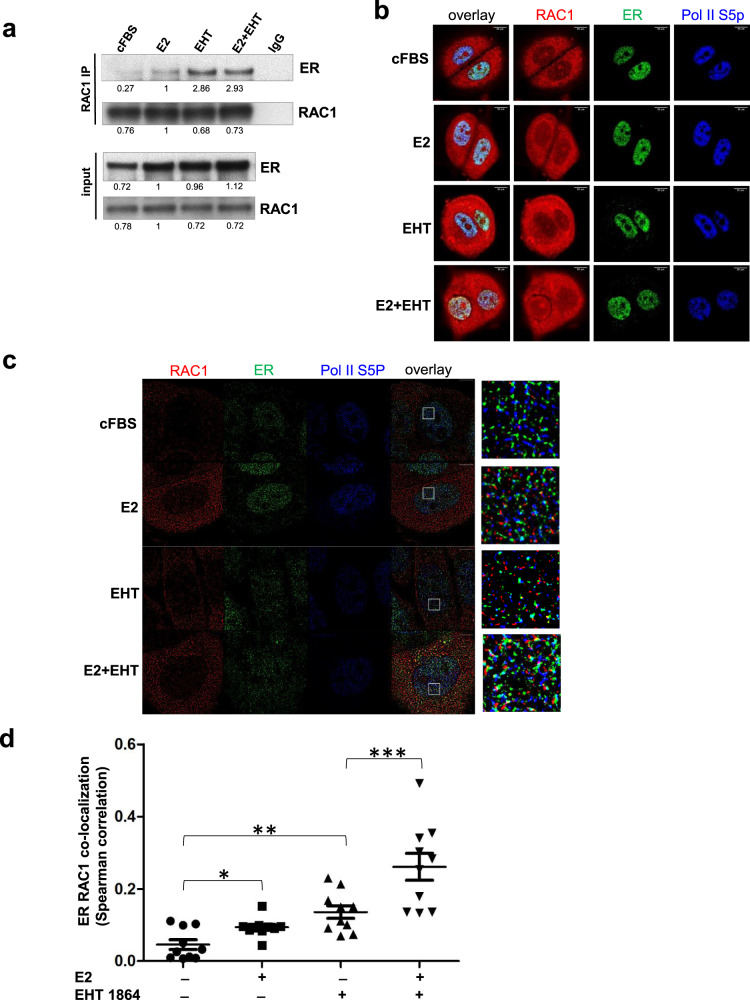
RAC1 interacts with ER. **A** MCF-7 cells were maintained in the estrogen-deprived media (cFBS) for three days followed by 1 h treatment of 10 nM E2, 50 µM EHT 1864, or 10 nM E2 plus 50 µM EHT 1864. Prepared nuclear lysates were subjected to immunoprecipitation with an antibody against RAC1.The input and immunoprecipitated samples were probed for ER or RAC1 using Western blot. Targets were quantified using Image Studio (Li-CoR). ER or RAC1 protein level in E2-treated sample from input or IP was set as 1 for comparison. **B**, **C** MCF-7 cells were grown on a cover slide and were treated as in (**A**). After 1 h treatment, the cells were fixed and stained with antibodies to RAC1, ER and RNA pol II S5P for confocal (**B**) or STORM imaging analysis (**C**). Targets are color-coded as shown at the top. Enlarged areas from selected regions inside nucleus (indicated by the square) are shown in the last column in (**C**). **D** The Spearman’s correlation coefficient for ER-RAC1 co-localization was calculated from 10 cell nuclei under the indicated treatment conditions. *P = 0.0032; **P = 0.0002; ***P < 0.0001 (Student’s *t* test).

To explore the possibility of a RAC1-ER multi-protein complex, we prepared nuclear extracts from estrogen-deprived MCF-7 cells that were treated with 10 nM E2, 50 µM EHT 1864 or their combination for 1 h and performed co-immunoprecipitation assays. E2, EHT 1864 or their combination increased nuclear portion of ER protein levels through fractionation (Fig. 4A), despite the total ER protein level was slightly reduced by EHT 1864 at 1 h (Fig. 2G). E2 stimulated the interaction between ER and RAC1 in nucleus by 2.7 fold after adjusting to the increased ER inputs. EHT 1864, in the absence or presence of E2, enhanced the association between ER and RAC1 to greater degrees (7–9 fold) (Fig. 4A). Thus, a RAC1-ER interaction is stabilized by the Rac inhibitor, even though it ultimately triggers ER degradation (Fig. 2G). We performed confocal microscopy to assess the localization of ER, RAC1 and RNA pol II-S5P, which acts as a nucleus marker, in MCF-7 cells (Fig. 4B). A large portion of RAC1 was associated with the cell membrane and the cytoplasm, but a small amount of RAC1 was seen in the nucleus. Exposure to E2 or EHT 1864 increased RAC1-ER co-localization in nucleus (Fig. 4B). We applied super-resolution Stochastic Optical Reconstruction Microscopy (STORM) [38] to investigate ER-RAC1 interaction in greater detail (Fig. 4C). E2 increased RAC1-ER co-localization in nucleus, as did EHT 1864, both in the presence and absence of E2 (Fig. 4C, D). It is of interest that ETH 1864 alone increased ER-RAC1 interaction to a similar level as E2 while the EHT1684 plus E2 increased more ER-RAC1 interaction inside nucleus (Fig. 4C, D). EHT1864 also increased the co-localization of RAC1-ER in cytoplasm (Fig. S2A). However, ER-RNA pol II S5P interaction did not change significantly under all treatment conditions (Fig. S2B). RAC1-RNA pol II S5P interaction increased only in cells treated with E2, but not in the presence of EHT 1864 (Fig. S2C). We also performed imaging studies using ER-negative MDA-MB-231 cells and MDA-MB-231 cells with stably expressed ER (Fig. S2D, E). We could detect, using confocal microscopy, strong ER signals in the nucleus of MDA-MB-231 cells with ER expressed, but not in MDA-MD-231 cells (Fig. S2D). From STORM imaging, significant RAC1 and RNA pol II S5P interaction could be detected only in MDA-MB-231 cells with ER stably expressed (Fig. S2E).

### RAC1 is recruited to the ER-binding loci at chromatin upon estrogen treatment

**Fig. 5:**
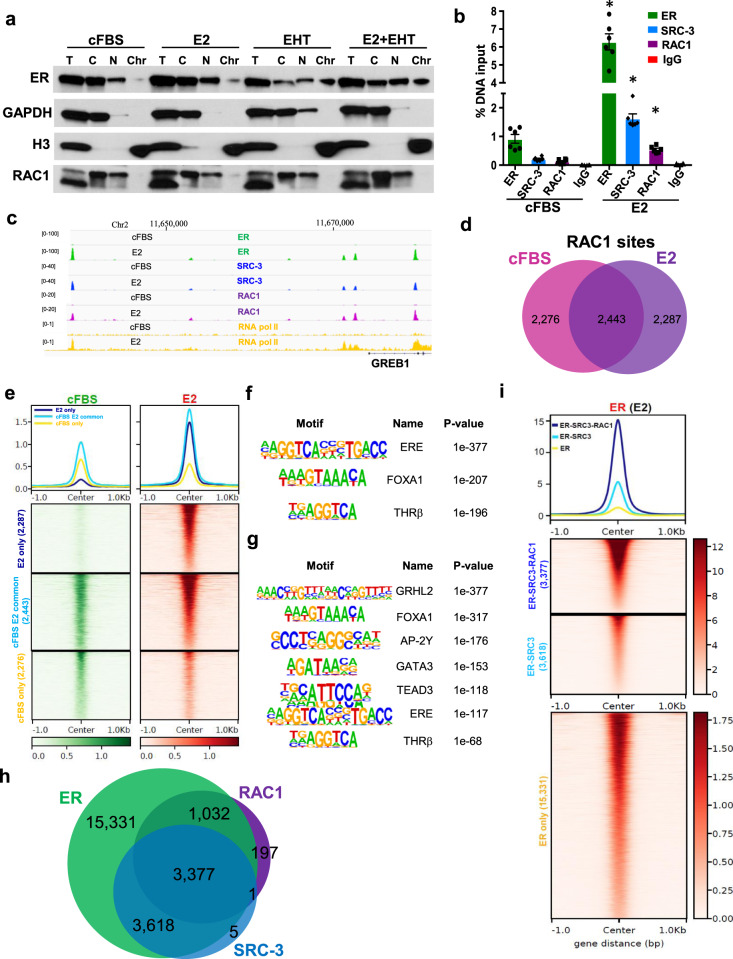
RAC1 co-localizes with ER on chromatin. **A** MCF-7 cells were treated as in Fig. 4A. The cell extract was fractionated into different portions and analyzed by Western blotting with the indicated antibodies. T total cellular lysate, C cytoplasmic fraction, N nuclear soluble fraction, Chr chromatin. **B** MCF-7 cells were cultured in estrogen-deprivation media for three days followed by the treatment with 10 nM E2 for 45 min. No E2-treated sample (cFBS) was served as a control. ChIP-qPCR analyses were performed for chromatin occupancies of ER, SRC-3, or RAC1, at a target region within the *GREB1* enhancer. *P < 0.0001, compared to each control sample (Student’s *t* test). **C** ChIP-seq analyses were performed for ER, SRC-3, RAC1, and RNA pol II, using MCF-7 samples prepared as in (**B**). Target occupancies at the *GREB1* enhancer region were shown in IGV genome browser tracks. **D** Venn diagram of identified RAC1 chromatin occupancy sites (derived from two biological replicate samples, *q* < 10^−10^) in the presence or absence of E2. **E** The average profiles and heatmaps of RAC1 signals at three groups of RAC1 occupancy sites as separated in (**D**). Homer motif analysis indicated enriched motifs at RAC1 sites only detected with E2 treatment (**F**) or motifs at RAC1 sites commonly detected with or without E2 (**G**). **H** Venn diagram of the overlapping of occupancy sites for ER, SRC-3 or RAC1 (derived from two biological replicate samples, *q* < 10^−10^) in the presence of E2. **I** The average profiles and heatmaps of ER signals in the presence of E2 at 3 groups of ER binding sites that were occupied by ER/SRC-3/RAC1, ER/SRC-3, or ER only.

We performed cellular fractionation assays to determine whether RAC1 inhibition affects the intracellular localization of ER. We detected increased nuclear and chromatin portion of ER after 1 h E2 treatment. Although RAC1 was mostly cytoplasmic, some RAC1 protein was present in nucleus. EHT 1864 treatment increased the amount of ER on chromatin in the presence of estrogen, but also in its absence (Fig. 5A). This was unexpected, as ER ligand is thought to be essential for promoting ER binding to chromatin. We failed to detect RAC1 on chromatin, which is likely due to indirect interaction between RAC1 and DNA (Fig. 5A).

To better define the chromatin localization of RAC1 and ER following estrogen exposure, we performed ChIP-qPCR analysis in MCF-7 cells that were first deprived of estrogen and then exposed to E2 for 45 min. Using antibodies against ER, SRC-3 (NCOA3), or RAC1, we observed that E2 rapidly increased the presence of all three proteins at an ER binding site within the *GREB1* enhancer region (Fig. 5B). The percentages of DNA recovery were different for each target, however, the fold changes upon E2 treatment were similar (Fig. 5B). Similar results were found in T47D cells (Fig. S3A).

We performed ChIP-seq analyses and found that estrogen stimulated the global co-localization of ER, SRC-3 and RAC1 on chromatin. Similar to the ChIP-qPCR results, we detected minimal signals for ER, SRC-3 or RAC1 within the *GREB1* enhancer region in the absence of E2, but saw dramatic increases in their signals at multiple loci in the presence of E2 (Fig. 5C). E2 also increased RNA Pol II localization at both enhancer and promoter regions of *GREB1* (Fig. 5C), consistent with its increased transcription. We also assessed the genome-wide association of these factors. As expected, E2 increased the chromatin association of ER at most of its binding sites; the same was true for SRC-3, albeit to a lesser extent (Fig. S4). RAC1 was found at numerous overlapping sites both in the absence and the presence of E2 (Fig. 5D). E2 increased overall RAC1 signals on chromatin (Fig. S4), specifically at the sites detectable only in the presence of E2 (Fig. 5D, E). RAC1 sites detected only in the presence of E2, that had low signals without E2 (Fig. 5E), are enriched for several motifs, such as estrogen response element (ERE), FOXA1 and half ERE (Fig. 5F). Common RAC1 sites detected in the absence and presence of E2, where E2 modestly increased RAC1 signals from higher basal levels (Fig. 5E), were enriched for additional motifs such as GRHL2, AP-2γ, GATA3, and TEAD3 (Fig. 5G). ERE motifs were not enriched at the RAC1 sites that were only identified in the absence of E2, where E2 did not increase the signals (Fig. 5D, E). The majority of E2-stimulated RAC1 occupancy sites (>95%) overlapped with ER-binding sites while 73.3% of the sites overlapped with SRC-3 occupancy sites (Fig. 5H). Intriguingly, the sites occupied by ER, SRC-3, and RAC1 represented the sites with the highest ER signals (Fig. 5I) while the sites occupied only by ER, had the lowest ER signals (Fig. 5I).

We annotated all detectable ER binding sites to 9317 genes, using homer software analysis. Among them, 432 genes (4.6%) were found to be stimulated by E2 (Fig. 3D). Similarly, we matched the sites occupied by ER/SRC-3/RAC1 (14.3% of all the ER sites), to 2131 genes, and found 247 genes (11.6%) that were stimulated by E2 (Fig. 3D and Supplementary Table 3). Thus, the sites occupied by ER/SRC-3/RAC1 represent functionally important sites associated with ER target genes.

### RAC1 inhibitor increases the chromatin association of RAC1 and ER

**Fig. 6:**
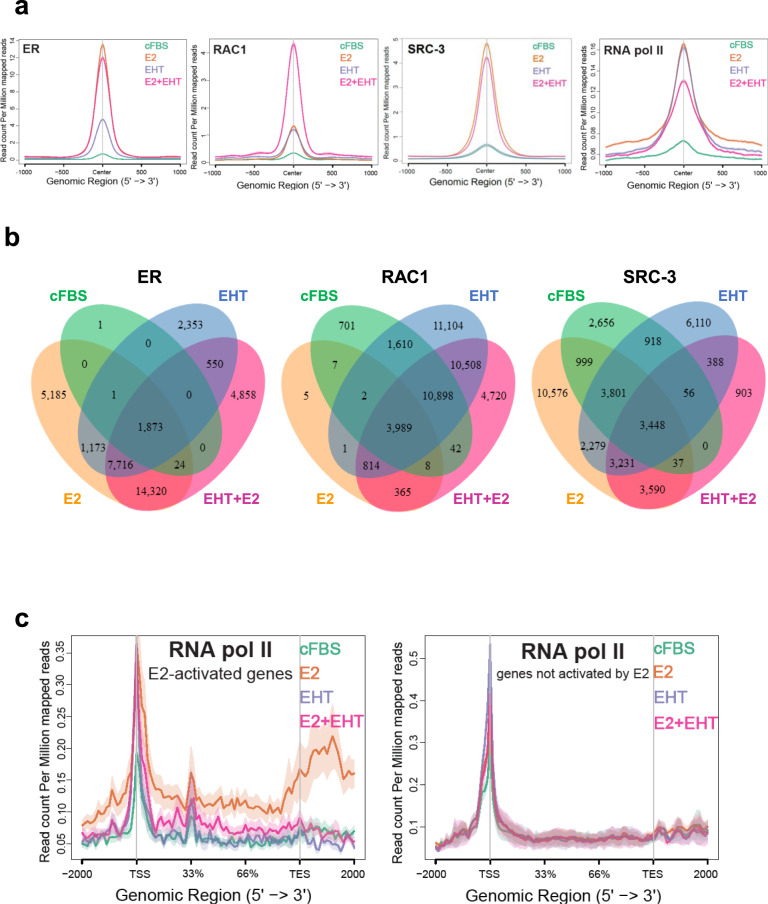
RAC1 inhibitor affects ER enhancer function. **A** Average binding profiles derived from ChIP-seq analyses for ER, RAC1, SRC-3, or RNA pol II in MCF-7 cells treated with vehicle control, 10 nM E2, 50 µM EHT 1864 or their combination for 45 min. The top 10,000 ER binding sites in the presence of E2 were selected for signal profiles of ER, SRC-3 and RNA pol II, while the top 1000 RAC1 sites were selected for RAC1 signal profiles. **B** The Venn diagrams of occupancy sites detected for ER, SRC-3 and RAC1 (*q* < 0.01) under the indicated treatments. **C** The average RNA pol II signal profiles for the indicated treatments at a group of E2-activated genes (left panel) and at a group of non ER target genes (right panel).

RAC1 interacts with ER and is present at the ER binding sites, which are consistent with the ability of the RAC1 inhibitor to decrease ER-activated transcription. To define this mechanistically, we performed more ER, SRC-3, RAC1 and RNA Pol II ChIP-seq in MCF-7 cells that were treated with E2, EHT 1864, or E2 plus EHT 1864. We treated cells with 50 µM EHT 1864 for 45 min and selected the top target occupancy sites to identify the primary effects of RAC1 inhibition. EHT 1864 increased E2-independent ER binding to chromatin (Fig. 6A), consistent with our results from cell fractionation assay (Fig. 5A) as well as STORM imaging (Fig. 4C, D). EHT 1864 also increased RAC1 occupancy on chromatin in the absence of E2, and to a much higher level in the presence of E2 (Fig. 6A) that were reflected in STORM imaging results (Fig. 4C, D). Intriguingly, SRC-3 was minimally affected, but RNA Pol II was increased, at the ER binding sites, by EHT 1864 in the absence of E2 (Fig. 6A). In the presence of E2, EHT 1864 did not increase more E2-induced occupancies of ER, SRC-3 or RNA pol II (Fig. 6A). Thus, EHT 1864 led accumulations of ER/RAC1 as well as RNA pol II complexes at the ER binding sites regardless of the presence of E2. EHT 1864 also changed the numbers of target occupancy sites detect for ER, RAC1, and SRC-3 (Fig. 6B) although the new sites identified showed weak signals (Fig. S4).

We selected a group of E2-induced genes to test RNA pol II response to E2 and EHT 1864 treatments (Fig. 6C). As expected, E2 increased RNA pol II level at transcriptional start sites (TSS), gene bodies and transcriptional termination sites (TES) of these ER target genes. EHT 1864 paused RNA pol II at the promoters of these genes, similar to the pattern at the ER binding sites (Fig. 6A), preventing RNA Pol II from moving across the gene bodies (Fig. 6C). For a group of active genes not regulated by estrogen, EHT 1864 exposure did not pause RNA Pol II at promoters (Fig. 6C). Thus, RAC1 activity is critical for releasing RNA pol II at the enhancers and promoters of ER target genes for productive RNA elongation.

## Discussion

While *RAC1* plays an essential role in mouse embryogenesis [39], genome-wide screens have found that *RAC1* could be knocked out in cells from several cancer types [40]. We have shown that *RAC1* is required for cell growth in ER-positive breast cancer cells, and that Rac inhibitors have more potent anti-proliferative effects on ER-positive than ER-negative breast cancer cells, as has been suggested by multiple studies before [28, 29]. In contrast to demonstrating active RAC1 enhances ER transcriptional activity [28], we found down-regulation of RAC1 leads to reduction in both ER protein level and activity. Moreover, we identified RAC1 is a critical component of the ER complex. The effects of RAC1 inhibition on ER protein stability and ER transcriptional activity appear to involve separate mechanisms. To mediate rapid ER degradation in MCF-7 cells, sufficient inhibition of RAC1 activity (requiring 25 µM or more EHT 1864) or > ~90% reduction of RAC1 protein needs to be achieved (Figs. 1B, 2G). However, EHT 1864 at 10 µM or lower was effective in blocking estrogen-stimulated gene expression (Fig. 3; Fig. S3B) and estrogen-promoted cell growth (Fig. 1D, E) [28]. Thus, the inhibition of ER transcriptional activity by EHT 1864 happens below the threshold required for ER protein degradation; this may be therapeutically relevant, since complete RAC1 inhibition is difficult to achieve in vivo and will likely be more toxic.

We focused on the primary effects of RAC1 inhibition. The analyses of newly synthesized RNA and microscopic imaging were performed in cells treated with EHT 1864 for 1 h. ChIP assays were performed after RAC1 activity was maximally inhibited for 45 min. Our results suggest that the interaction between active RAC1 and ER is transient without estrogen, but is required to maintain ER protein levels. ER transcriptional activity was inhibited by EHT 1864 within 1 h when changes in the MAPK or PI3K signaling pathway were not detected, suggesting the direct effect of RAC1 activity on ER transcriptional activity. However, prolonged treatment with RAC inhibitor affects multiple signaling pathways that interact with ER signaling, and leads to decreased ER protein and chromatin occupancy [28].

We found a small portion of RAC1 present in the nucleus of MCF-7 cells (Figs. 4A–C, 5A). A nuclear localization signal was identified at the C-terminus of RAC1 [41]. Nuclear RAC1 can modulate actin polymerization, control nuclear membrane shape and cell invasiveness [42]. RAC1 may also be transported into nucleus with the ER complex. ER directs the chromatin association of RAC1 to the ER binding loci. In an ER-negative MCF-7 derivative cell line [43], RAC1 was not found at the loci occupied by ER in MCF-7 cells (Fig. S5A, B). But when ER was stably expressed in ER-negative MBA-MD-231 cells, we could detect RAC1 was present at the same sites bound by ER (Fig. S5A, C).

RAC1 can regulate transcription with several transcription factors, such as β-catenin/TCF-regulated Wnt target genes, and RORγt-regulated IL17A [44–46]. Here, we have shown that RAC1 can function within the ER complex to regulate ER target genes. To determine whether this regulation can be more broadly applied to the activity of other steroid hormone receptors, which share common co-activators, we examined whether androgen can similarly stimulate the chromatin association of RAC1. Indeed, we found androgen increased RAC1 at the loci occupied by the androgen receptor (AR) in MCF-7 cells (Fig. S5D, E). Therefore, RAC1 appears to be a common cofactor for steroid hormone receptors. Many more transcription factors or TF complexes may depend on RAC1 for their transcriptional activity.

RAC3 has been suggested as a cofactor for ER [47]. However, RAC3 is expressed at a much lower level than RAC1 (Fig. S1C, D) in MCF-7 cells and we failed to detect the association of RAC3 with the ER complex on chromatin using ChIP-qPCR or ChIP-seq assays. The sequence of a peptide identified to interact with ER through a phage display screen [47] is present in RAC1, which suggests that the ER-RAC1 interaction could be direct. We conducted our experiments using endogenous RAC1, rather than relying on overexpression or reporter gene assay [47]. The C-terminal nuclear localization sequence in RAC1 [41], which is not present in RAC2 or RAC3, may be critical for chromatin function of RAC1.

**Fig. 7:**
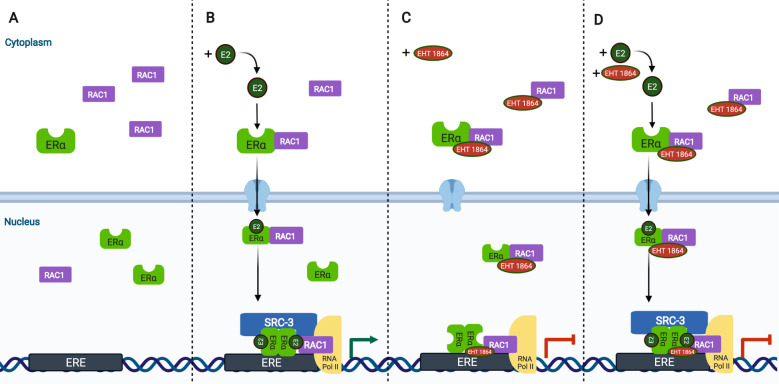
The model of RAC1 on ER transcriptional activity. **A** In the absence of estrogen, there is little ER-RAC1 complex on chromatin. **B** Estrogen-bound ER dimer brings its coactivator SRC-3, RAC1 as well as RNA pol II to ER binding sites to activate gene transcription. **C** In the presence of RAC1 inhibitor only, ER-RAC1 complex is stabilized on chromatin; RNA pol II is accumulated at ER binding sites and promoters of ER target genes. **D** In the presence of both estrogen and RAC1 inhibitor, ER complex including SRC-3 and RAC1 is stabilized on chromatin; RNA pol II, which fails to activate gene transcription, is accumulated at ER binding sites and promoters of ER target genes. ER transcriptional activity is blocked by RAC1 inhibitor, which is distinct to anti-estrogens and prevents the interaction between ER and its coactivator SRC-3. RAC1 inhibition provides an alternative way to attenuate ER transcriptional activity. The figure was created with BioRender.com.

Distinct from endocrine therapies, RAC1 inhibition blocks RNA Pol II activity at the ER-regulated enhancers and promoters (Fig. 7). Thus, our work paves the way for the development of novel RAC1 inhibitors that could be used to treat endocrine-resistant, ER-positive breast cancer in the clinic. It is also possible that RAC1 inhibitors may be valuable in treating other diseases, such as AR-positive prostate cancer.

## Materials and methods

### Cell line, compounds and antibodies

EHT 1864 was purchased from APExBIO (B5487). 17-β-estradiol (E2) (E2758), 4-hydroxytamofen (OHT) (H7904), Fulvestrant (I4409), and 5-bromouridine (850187) were purchased from Sigma. MCF-7 cells were maintained in Dr. Marc Lippman’s laboratory. Other cell lines were from ATCC. Cell lines were authenticated with short tandem repeat profiling and were regularly tested for mycoplasma in house. MCF-7 cells were cultured in IMEM (Invitrogen) with 5% fetal calf serum (FBS) (Hyclone). Other cells were maintained in DMEM (Invitrogen) with 10% FBS. For estrogen deprivation, the cells were shifted to IMEM containing 5% charcoal-treated FBS (cFBS) (Hyclone) for three days. The antibodies against ER (sc-543 and sc-8002) and SRC-3 (sc-7216) were from Santa Cruz Biotechnology. The antibodies against RAC1 were from ThermoFisher Scientific (PA1091) and Millipore (clone 23A8, 05-389). RAC3 antibody was from Abcam (ab129062). AR antibody was from Millipore (06-680). Anti-BrdU was from BD Biosciences (555627).

### Cell proliferation assay

Cells were seeded (5000/well) in 96-well white plate and incubated overnight at 37 °C before treatment with EHT 1864. Cell viability was measured with PrestoBlue cell viability reagent (ThermoFisher Scientific). Dose response curves from at least triplicate measurements were generated using GraphPad Prism software. For colony assay, the cells were treated in 24-well plate followed by staining with 1% crystal violet or cell counting using Moxi Z mini automated cell counter (Orflo Technologies).

### siRNA transfection

siRNA transfection were performed using Lipofectamine RNAiMAX transfection reagent (ThermoFischer Scientific #13778150). Predesigned siRNAs were from ThermoFisher Scientific: siRNAs against RAC1 (#4390826 S11711 and S11713), ESR1 (#4392420 S4823 and S4835), RAC3 (#4392420 S11717) and negative control no.1 siRNA (AM4635).

### Co-immunoprecipitation and western blot

Co-immunoprecipitation (co-IP) was performed as described [48] using nuclear lysates purified with subcellular protein fractionation kit (ThermoFisher Scientific, 78840). The samples were run on a 4–12% premade polyacrylamide gel (Invitrogen).

### Chromatin immunoprecipitation (ChIP) and ChIP-seq

ChIP and ChIP-seq were performed as described [49]. The sequencing reads were aligned to the human genome (hg19) using bowtie2 (2.2.6) [50] with the default parameters. The peaks were called with MACS2 (2.1.1.20160309) [51]. Motif analysis was performed using HOMER (4.11.1) [52]. DeepTools (3.1.3) [53] or NGS plot (2.61) [54] were used to generate heatmaps and average binding profiles.

### RT-qPCR and RNA-seq

Total RNA samples were prepared with Trizol (ThermoFisher Scientific #15596018). cDNA was made with qScript cDNA supermix (Quantabio #95048-100). Real-time PCR analysis was performed with IQ SYBR Green supermix (Bio-Rad #170-8886). The primers are listed in Supplementary Materials. RNA-seq libraries were made with NEBNext rRNA depletion kit (#E6310) and NEBNext Ultra II directional RNA library prep kit (#E7760). For newly synthesized RNAs, cells were treated in the presence of 2 mM 5-bromouridine (BrU) (Sigma #850187) for 1 h. Ten µg of total RNA samples were immunoprecipitated at 4 degree for 1 h in PBS containing 0.5% BSA, 0.05% Tween-20, RNA inhibitor (NEB #M0314L), 2 µg of mouse anti-BrdU (BD Biosciences #555627) and 30 µl of goat anti-mouse IgG magnetic beads (NEB #S1431S). Fifty pg of BrU-labeled firefly luciferase RNA was added as a spike-in control, which was generated from in vitro transcription with addition of 5-bromouridine 5’-triphosphate (Sigma #B7166) using HiScribe T7 quick high yield RNA synthesis kit (NEB #E2050S). After two rounds of RNA-IP, the purified RNAs were subjected to RT-qPCR or RNA-seq analysis. The sequencing reads were aligned to human genome (hg19) using Tophat (2.1.0) [55]. Salmon [56] or featureCounts (2.0.1) [57] was used to count total RNA-seq or BrU-labeled RNA-seq samples. GSEA (3.0) [58] was used to perform gene set enrichment analysis and edgeR (3.24.1) [59] was used for differential gene expression analysis.

### Immunofluorescence

MCF-7 cells were treated for 1 h after seeded onto a 4-well glass bottom slide (ibidi # 80427) in IMEM-5% cFBS for three days. Cells were fixed with 3% paraformaldehyde and 0.1% glutaraldehyde for 10 min and treated with 0.1% NaBH4 in PBS for 7 min. Cells were permeabilized for 15 min in 0.5% Triton X100/PBS, and blocked for 90 min in 5% normal goat serum, 0.3% Triton X100/PBS. Cells were incubated with the primary antibodies overnight at 4 °C, washed five times with 1% normal goat serum, 0.05% Triton X100/PBS. Alexa fluor 488-, Alexa fluor 568- (Invitrogen) or Janelia 645-labeled secondary antibody (1:1000) was added for 30 min at room temperature. After five times wash, the cell samples were imaged with a confocal microscope Nikon C2.

### Stochastic optical reconstruction microscopy (STORM) imaging

STORM imaging was carried out as described [60]. Samples were imaged continuously for 10,000 frames per filter range at a frequency of 20 ms. Localization analysis was carried out with Image J version 1.53b thunderstorm plugin version 2016-09-10. A wavelet filter was used prior to utilizing a weighted least squares fitting method for localization of molecules. Molecule list files were further analyzed using Coloc-Tesseler software [61]. Regions of interest were manually selected for nucleus and Spearman correlation was calculated per image.

### Statistical analysis

Significance was calculated using unpaired, two-tailed Student’s *t* test.

## Supplementary information

Supplementary materials

Supplementary Figure 1

Supplementary Figure 2

Supplementary Figure 3

Supplementary Figure S4

Supplementary Figure 5

Supplementary Table 1

Supplementary Table 2

Supplemental Table 3

## Data Availability

RNA-seq and ChIP-seq data are deposited under GEO accession number GSE169096.
