# Supplementary material for: RAC1 plays an essential role in estrogen receptor alpha function in breast cancer cells
Source: Oncogene. 2021 Aug 9;40(40):5950–62. doi: 10.1038/s41388-021-01985-1 (PMC8497275; doi:10.1038/s41388-021-01985-1)
Supplement: Supplementary file 1 — Supplementary materials [file 41388_2021_1985_MOESM1_ESM.docx]

**Table** Primers used for RT-qPCR analysis

| **target gene** | **forward primer** | **reverse primer** |
| --- | --- | --- |
| *RAC1* | ACAGATTACGCCCCCTATCCT | AATGATGCAGGACTCACAAGG |
| *ESR1* | ATCCTGATGATTGGTCTCGTCT | GGATATGGTCCTTCTCTTCCAGA |
| *GREB1* | ATCAGCTGCTCGGACTTGCTG | TGAGCTCCGGTCCTGACAGATG |
| *TFF1* | GCGCCCTGGTCCTGGTGTCCAT | GAAACCACAATTCTGTCTTTCAC |
| *CXCL12* | TTCTCAACACTCCAAACTGTGC | TCCAGGTACTCCTGAATCCACT |
| *CCND1* | TCCTGTCCTACTACCGCCTCAC | CCTCCTCCTCTTCCTCCTCCT |
| *MYC* | GAGTCTGGATCACCTTCTGCTG | AGGATAGTCCTTCCGAGTGGAG |
| *IL1R1* | GAGCGGCAGGAATGTGACAA | CAGGAGGGAGTCACCATCTTCA |
| *TNFRSF11B* | CCACAATGAACAACTTGCTGTG | CAGCTGATGAGAGGTTTCTTCG |
| *GAPDH* | GAAGGTGAAGGTCGGAGTC | GAAGATGGTGATGGGATTTC |

**Supplementary figure legends**

**Figure S1. RAC1 in breast cancer cells.** (A) T47D cells were transfected with two different siRNAs targeting *RAC1* or *ESR1,* respectively. Non-targeting siRNA was used as a control. Cell lysates were collected 3 days after transfection for Western blot analysis. Targets were quantified as in Fig.1B. (B) Western blot analysis for RAC1 protein in MDA-MB-468 cells with expression of Cas9, or a derived cell clone with expression of a guide RNA targeting *RAC1*. (C) Genome browser tracks and (D) the average mRNA expression levels for RAC family genes in MCF-7 cells detected within two RNA-seq data sets. (E) MCF-7 cells were transfected with siRNAs targeting *RAC1*, *ESR1* or *RAC3*, respectively. Cell lysates were collected after 1 day or 2 days. Western blot analysis was performed for ER, RAC1 or RAC3, with quantification performed as in Fig.1B. (F) Gene expression analysis was performed using RT-qPCR in MCF-7 cells that were transfected with siRAC3 for 2 days. Data were presented for two triplicate experiments as mean ± SD. (G) Proliferating MCF-7 cells were treated with the indicated conditions for 6 h before collecting cell lysates for Western blot analysis for ER.

**Figure S2. Imaging analyses for RAC1 and ER.** (A) MCF-7 cells were processed as in Fig.4C. Coloco-Tesseler STORM images are shown for each treatment condition with co-localized ER and RAC1 molecules shown in red, isolated ER or RAC1 molecules in blue or yellow, respectively. The Spearman’s correlation coefficient for ER-RNA pol II S5P (B) or RAC1-RNA pol II S5P (C) co-localization was calculated from 10 cell nuclei under the indicated treatment conditions as. *** *P* < 0.0001 (Student’s t test). MDA-MB-231 cells or MDA-MB-231ER cells (with stably expressed ER) were fixed and stained with antibodies to RAC1, ER and RNA pol II S5P for confocal imaging (D) or STORM imaging (E).

**Figure S3. RAC1 regulates ER target gene in T47D cells.** (A). T47D cells were hormone-deprived for 3 days before the cells were treated for 45 min with 10nM E2, 50µM EHT 1864, or their combination. The cell samples were processed for ChIP-qPCR assay for ER or RAC1, as in Fig.5B. Data were collected from triplicate assays and presented as percentage input DNA for each IP. Normal IgG was included for each condition. * *P* = 0.026; ** *P* = 0.008, compared to each control (Student’s t test). (B) T47D cells were hormone-deprived for 3 days before treating the cells with 10nM E2, 1µM OHT, 10µM EHT 1864 or the combination for 4 h. Total RNA samples were prepared for RT-qPCR analysis. *GREB1* expression levels after various treatments were compared to non-treated cells after normalization to *GAPDH* that was used as an internal control. Triplicate assays were performed and were presented as mean ± SD. * *P* = 0.017 (Student’s t test).

**Figure S4. Chromatin occupancies of ER, SRC-3 and RAC1 in MCF-7 cells.** The average signal profiles and heatmaps of ChIP-seq enrichment for ER, SRC3, and RAC1, in MCF-7 cells that were treated for 45min under four different conditions (control, 10nM E2, 50µM EHT 1864 or 10nM E2 plus 50µM EHT 1864). The target occupancy sites detected under all treatment conditions using MACS2 (q < 0.01) are included.

**Figure S5. RAC1 is co-localized with ER or AR on chromatin.** (A) Genome browser tracks are shown for ER or RAC1 ChIP-seq at the GREB1 enhancer region from the cells as indicated. (B) The average signal profiles for RAC1 ChIP-seq in MCF-7 cells or ER-negative MCF-7 C4-12 cells are shown at the ER/RAC1 occupancy sites detected in MCF-7 cells. (C) The average signal profiles for ER or RAC1 ChIP-seq in MDA-MB-231 cells with ER stably expressed. (D) MCF-7 cells were hormone deprived for 3 days and then treated with 100nM dihydrotestosterone (DHT) for 45 min. ChIP-seq analyses were performed for androgen receptor (AR) and RAC1. Genome browser tracks for *TFF1* locus (D) or the average signal profiles for AR and RAC1 at the AR occupancy sites are shown (E).
