# Supplementary figures and images for: RAC1 plays an essential role in estrogen receptor alpha function in breast cancer cells
Source: Oncogene. 2021 Aug 9;40(40):5950–62. doi: 10.1038/s41388-021-01985-1 (PMC8497275; doi:10.1038/s41388-021-01985-1)

Fig. S1.

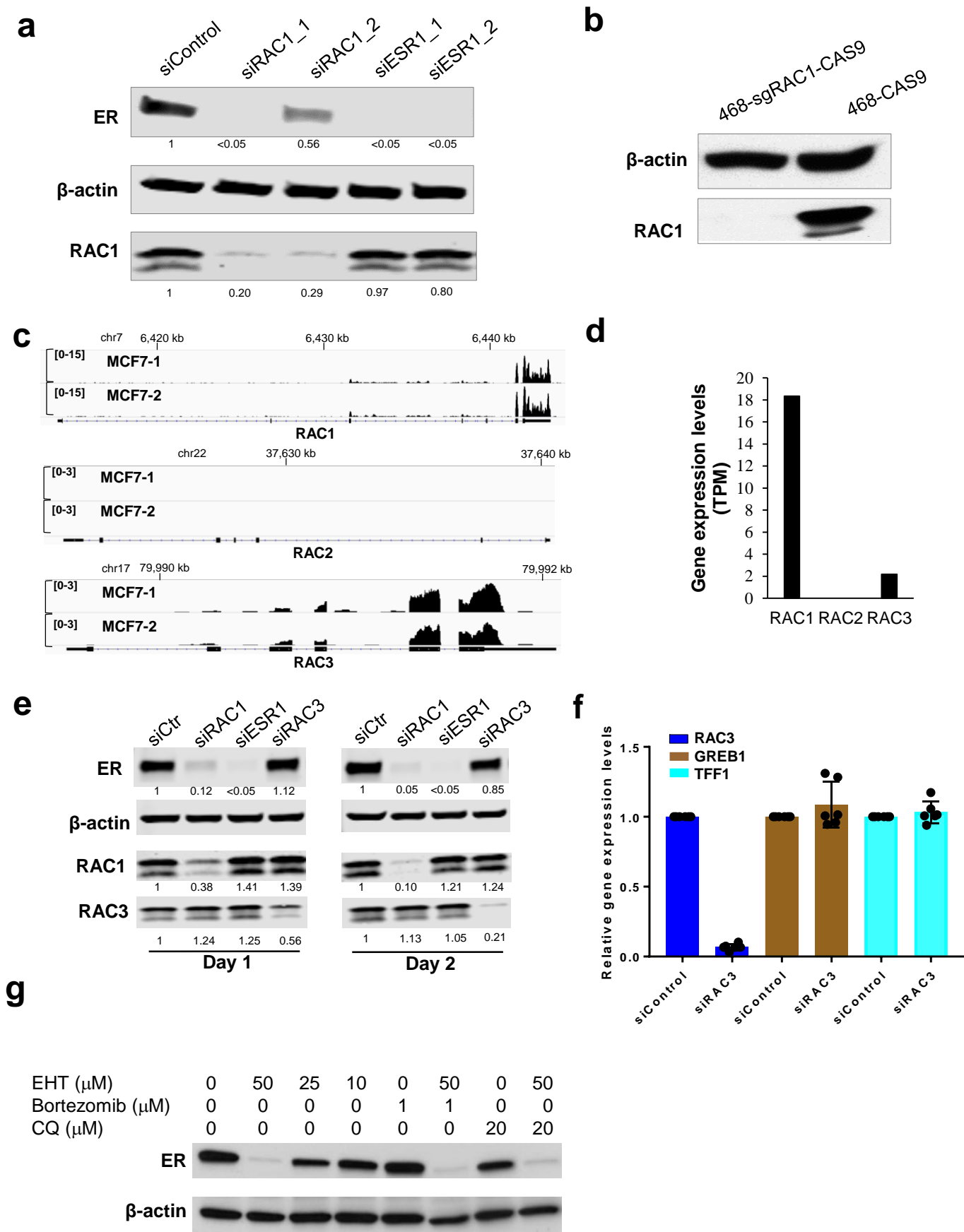

Supplement: Supplementary file 2 — Supplementary Figure 1 [file 41388_2021_1985_MOESM2_ESM.pdf]

Fig. S2.

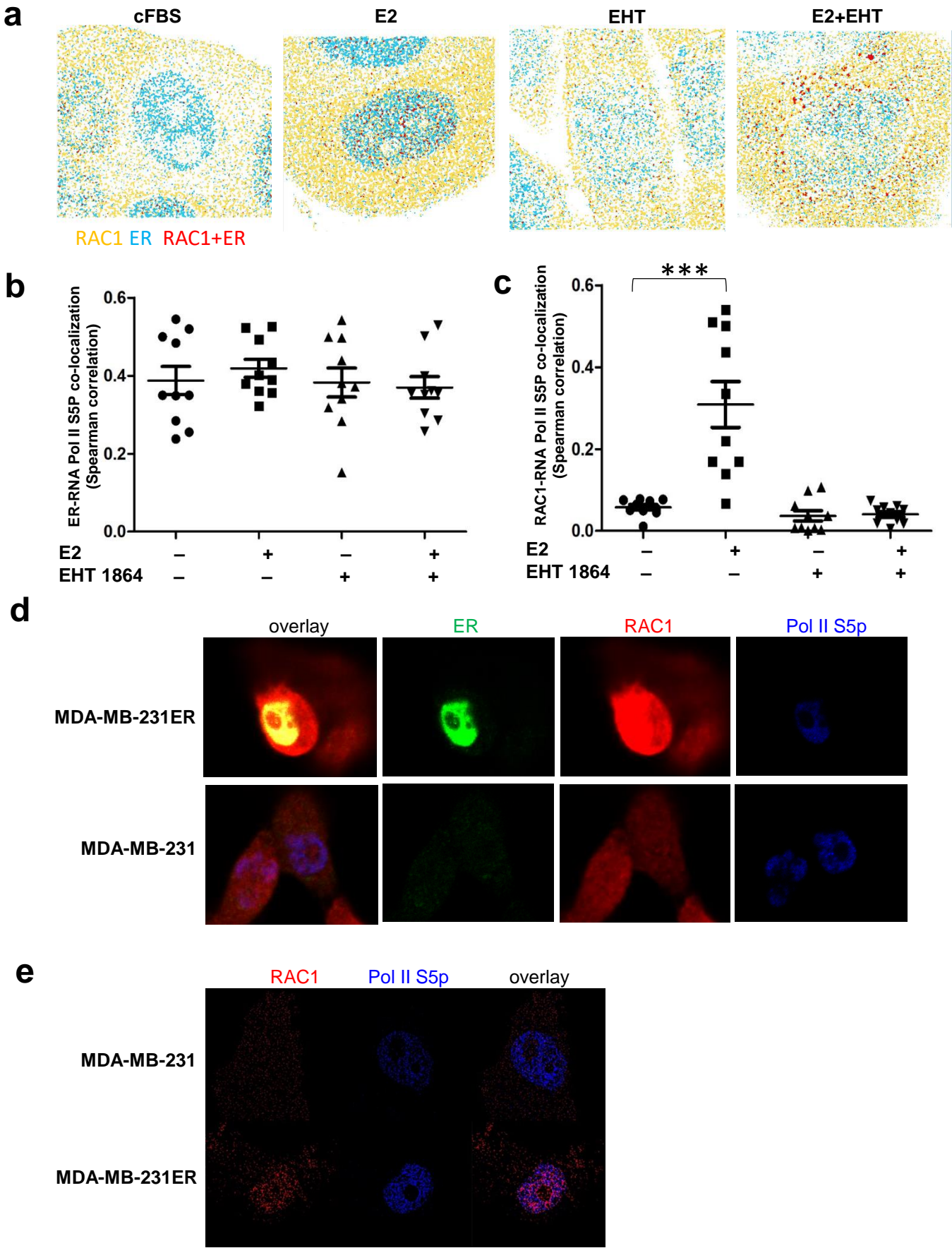

Supplement: Supplementary file 3 — Supplementary Figure 2 [file 41388_2021_1985_MOESM3_ESM.pdf]

Fig. S3.

a

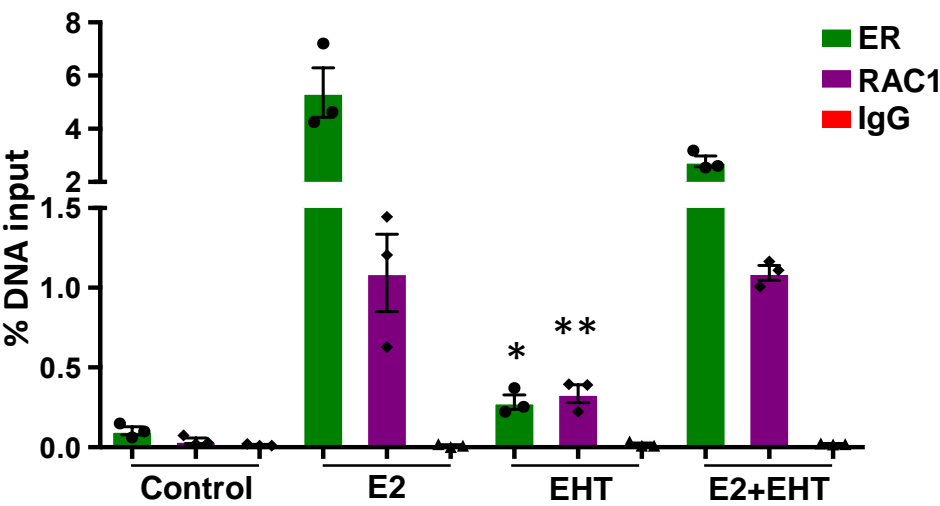

b

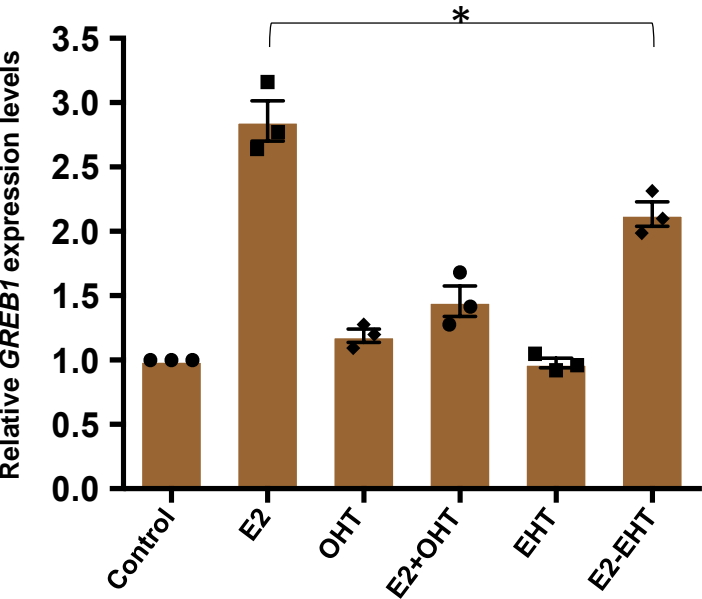

Supplement: Supplementary file 4 — Supplementary Figure 3 [file 41388_2021_1985_MOESM4_ESM.pdf]

Fig. S4.

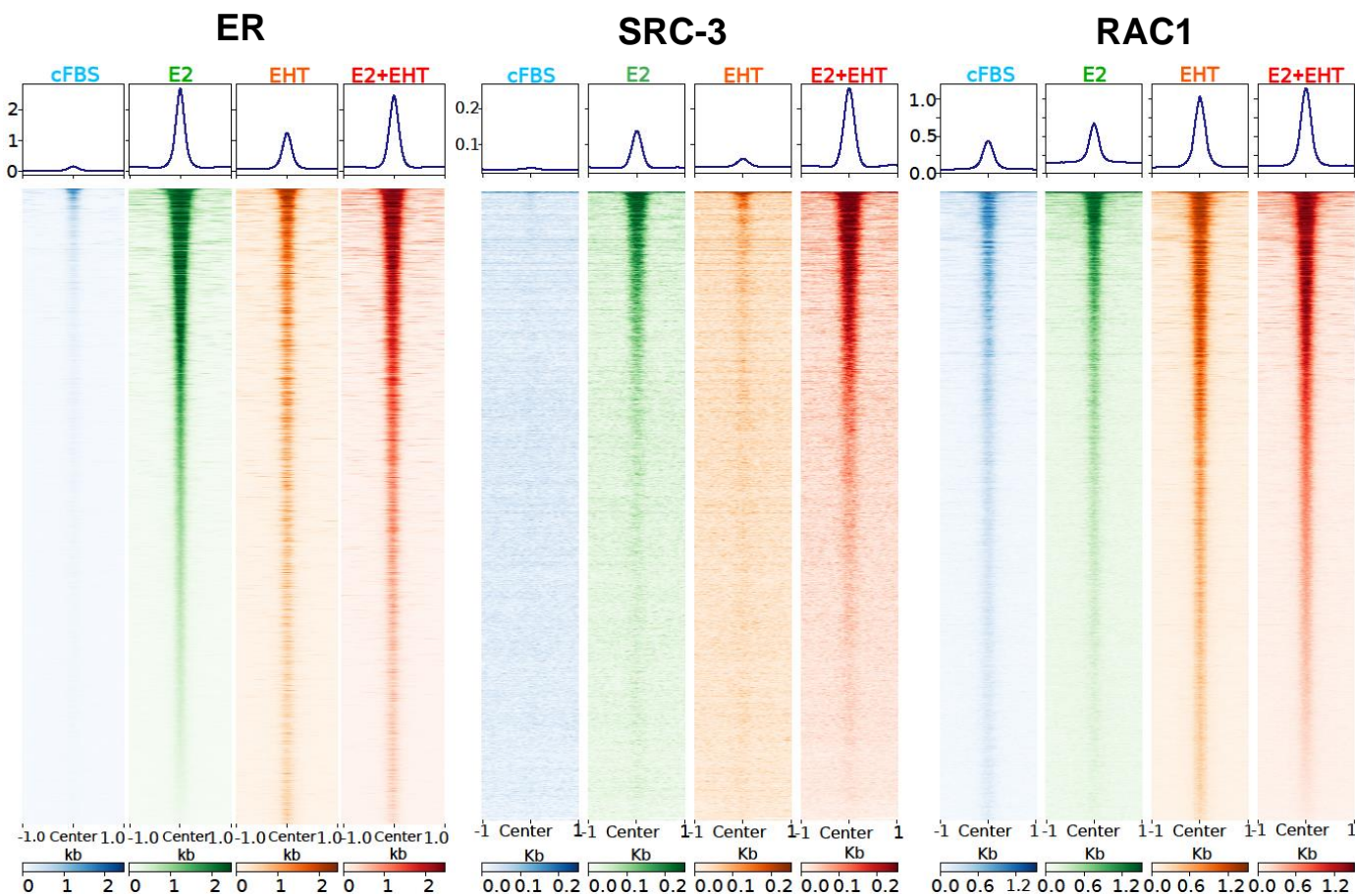

Supplement: Supplementary file 5 — Supplementary Figure S4 [file 41388_2021_1985_MOESM5_ESM.pdf]

**Fig. S5.**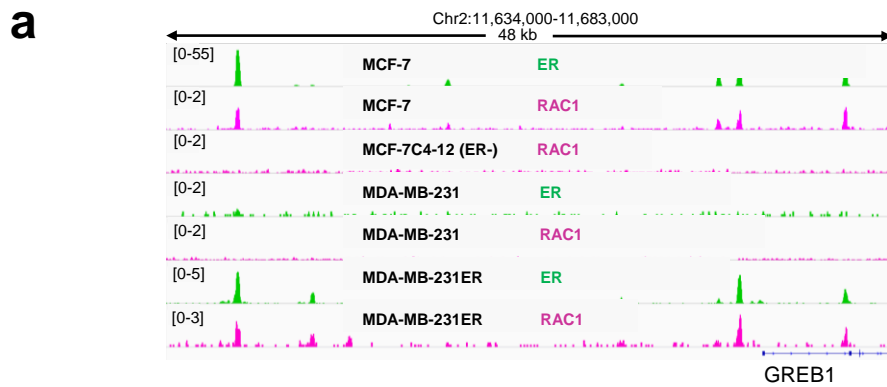

**b**

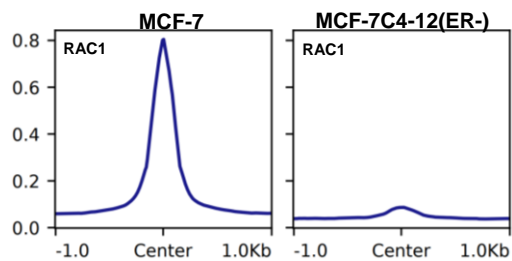

**c**

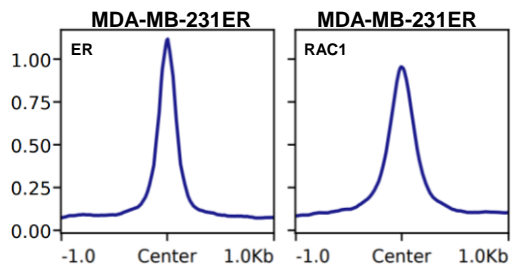

**d**

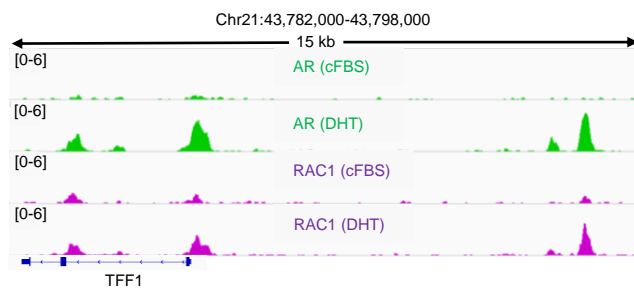

**e**

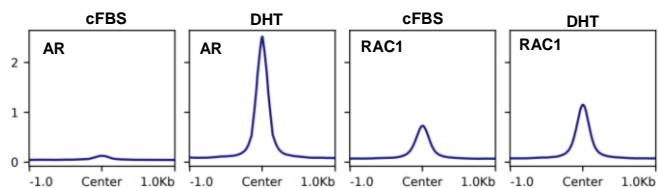

Supplement: Supplementary file 6 — Supplementary Figure 5 [file 41388_2021_1985_MOESM6_ESM.pdf]
